# Cord blood IgG for respiratory syncytial virus and subsequent infection during the COVID-19 pandemic

**DOI:** 10.1097/MD.0000000000041110

**Published:** 2025-01-03

**Authors:** Ritsuko Ogasawara, Mitsuyoshi Urashima

**Affiliations:** a Division of Molecular Epidemiology, The Jikei University School of Medicine, Tokyo, Japan; b Department of Obstetrics and Gynecology, The Jikei University School of Medicine, Tokyo, Japan; c Department of Pediatrics, The Jikei University School of Medicine, Tokyo, Japan.

**Keywords:** cord blood IgG, COVID-19, epidemiology, respiratory syncytial virus

## Abstract

The resurgence of respiratory syncytial virus (RSV) infection among Japanese infants during the coronavirus disease 2019 pandemic might be due to a decrease in cord blood anti-RSV immunoglobulin G (IgG) positivity resulting from reduced maternal RSV exposure. This study examined changes in the positivity before and during the pandemic to clarify the relationship between this positivity and infantile/severe RSV infections. Data from a prospective cohort study conducted in Tokyo, Japan, involving mother–child pairs of infants born between February 2019 and August 2022 were reanalyzed. Cord blood anti-RSV IgG levels measured by enzyme-linked immunosorbent assay were classified as positive, gray zone, and negative. We examined the relationship between antibody positivity and infantile (≤12 months old) and/or severe RSV infections as diagnosed by pediatricians. A total of 319 families participated. There was a significant decrease in cord blood anti-RSV IgG positivity from 65.1% in 2019 to 50.9% in 2022 (*P* = .02). Among infants followed up until their 2nd birthday, 43 (33.1%) were diagnosed with RSV infections. Of these cases, 5 were infantile/severe and occurred among the 50 infants classified as negative or gray zone, while none were observed among the 80 infants classified as positive (*P* = .004). Infantile/severe RSV infection was identified in 4 (16.7%) born during the pandemic, representing a significantly higher rate than 1 (0.9%) born before the pandemic (*P* < .001). This study revealed an association between lower cord blood anti-RSV IgG positivity during the coronavirus disease 2019 pandemic compared to before the pandemic, and an increased risk of subsequent infant/severe RSV infections.

## 1. Introduction

Declaration of coronavirus disease 2019 (COVID-19) as a pandemic by the World Health Organization (WHO) in March 2020 prompted stringent interventions, such as mask-wearing and social distancing, resulting in a notable decline in respiratory viral infections, such as respiratory syncytial virus (RSV) and influenza.^[1–3]^ Unexpectedly, after over a year-long decline in infections, an off-season resurgence of RSV emerged in infants and young children.^[4–6]^ Generally, RSV leads to substantial morbidity and mortality in infants and small children,^[7–9]^ sometimes with a more severe course than COVID-19 in this age group.^[10,11]^ Conversely, a comparable resurgence in influenza was not observed.^[12]^ Additionally, there was a rise in severe hepatitis cases in children in 2022, partly associated with adenovirus,^[13,14]^ although its correlation with pandemic measures was unclear.

During the pandemic, due to robust COVID-19 measures, pregnant women might have experienced a lower likelihood of exposure to these viruses compared with pre-pandemic years, leading to reduced levels of antivirus immunoglobulin G (IgG) antibodies in them. Hence, since IgG is transmitted from mothers to fetuses through the placenta,^[15]^ it is plausible that antiviral IgG levels in cord blood at birth might have been lower during the pandemic, potentially contributing to subsequent out-of-season outbreaks. This study aimed to compare cord blood anti-RSV IgG positivity at birth in infants born between the pre-pandemic year of 2019 and the pandemic year of 2022, investigating whether a decrease in positivity is linked to an elevated risk of infantile or severe RSV infections. Similar analyses were also conducted for influenza A and adenovirus, for comparative purposes.

## 2. Materials and methods

The Ethics Committee of the Jikei University School of Medicine approved this study. The committee waived the requirement for informed consent, since consent for collecting cord blood samples and their subsequent use had already been obtained through consent forms in the original studies, which are still ongoing. These original studies were previously approved by the same ethics committee. The study followed the reporting guidelines outlined in Strengthening the Reporting of Observational Studies in Epidemiology (STROBE).

### 2.1. Study design

This re-analysis of a prospective cohort study, which took place at Jikei University Hospital in Tokyo, Japan, involved mother–infant pairs from 2 concurrent studies: the randomized controlled trial “Primary prevention of food allergy by restricting maternal intake of processed meat and others during the first month after birth (Cohort I: ABC II),” and a prospective cohort study examining soluble PD-L1 levels in blood, to predict hypertensive disorders of pregnancy and pregnancy complications (Cohort II). Details on recruitment and procedures for each cohort are provided (see Methods S1, Supplemental Digital Content, http://links.lww.com/MD/O244 which illustrate the study cohort descriptions).

For Cohort I, pregnant women and their husbands, most of whom were first-time parents, were informed about the study during childbirth classes in the third trimester. In Cohort II, participants were informed about the study during antenatal checkups in the first or second trimester. Infants born between February 4, 2019 and December 31, 2021 were extracted from Cohort I, while infants born between January 1, 2022 and August 31, 2022 were extracted from Cohort II.

### 2.2. Participants

Of the 383 participants born between February 4, 2019 and August 31, 2022, this study included those born after 36 weeks of gestation and with available cord blood plasma samples. For twin births, only the firstborn of each pair was included in the analysis.

### 2.3. Cord blood plasma IgG positivity

Cord blood plasma samples were collected at birth and stored at −80 °C after complete deidentification. Plasma IgG levels were measured using a qualitative enzyme-linked immunosorbent assay (ELISA) targeting well-established immunodominant epitopes. This assessment used the following viral target kits from Abcam, Cambridge, MA: Anti-RSV Human IgG ELISA Kit; catalog no. ab108765, Anti-Influenza virus A IgG Human ELISA Kit; catalog no. ab108745, and Anti-Adenovirus IgG Human ELISA Kit; catalog no. ab108705. The following cutoff points specified in the measurement kit protocol were applied: positive (>11 standard units [SU]), negative (<9 SU), and gray zone (9–11 SU). Each sample was assessed once.

### 2.4. Incidence of RSV, influenza A, and adenovirus infection

Diagnoses of infectious diseases were established by local private practice pediatricians when infants were symptomatic through the utilization of rapid diagnostic kits or contextual evidence, including family infection history or nursery outbreaks, along with distinct clinical symptoms associated with each infection. The incidence of RSV, influenza A, adenovirus, and other diseases among participants up to their second birthday was thoroughly assessed through detailed interviews conducted by pediatricians engaged in the ABC II study (Cohort I) during outpatient visits at the infants’ second birthdays. Infants in Cohort II were excluded from the analysis of infection incidence because the follow-up of infants ended at 1 month of age.

In this study, “infantile/severe” RSV infection was defined as onset before the first birthday and/or meeting any of the following criteria: (1) persistent asthmatic bronchiolitis lasting over 1 month, (2) requiring emergency medical care with treatments such as intravenous therapy or steroid administration, (3) presenting tachypnea or low oxygen levels (SpO_2_ < 95%), 4) needing mechanical ventilation, or (5) resulting in death. Hospitalization was not included as a criterion for severity due to significant fluctuations in admission criteria resulting from the COVID-19 pandemic’s regional and temporal variations. All RSV cases not meeting the “infantile/severe” criteria were categorized as “mild.”

### 2.5. Surveillance data

Weekly counts of RSV, influenza, and cases with pharyngoconjunctival fever probably related to adenovirus infection were obtained from the Tokyo Metropolitan Infectious Disease Surveillance Center.^[16]^ The data were categorized into 3 age groups: 0 to <6 months, 6 to <12 months, and 12 to <24 months. The datasets spanned the period from January 1, 2018 to July 31, 2023.

### 2.6. Statistical analysis

Participants’ characteristics were compared between infants born between 2019 and 2022 by year. The chi-square test was used to compare anti-RSV IgG positivity between participants born in 2022 vs 2019. Further, sensitivity analysis was conducted, excluding mother–newborn pairs with siblings < 2 years old at the time of the study infant’s birth. This exclusion was based on the understanding that, since most children are infected with RSV by the age of 2 years,^[17]^ infants with siblings < 2 years old were considered to be at a higher risk for RSV infection compared to those without such siblings. Additionally, associations between anti-RSV IgG positivity and the risk of RSV infection before the second birthday were estimated using risk differences and 95% confidence intervals. Participants were divided into infants born pre-pandemic and those born during the pandemic, with a cutoff date of March 11, 2020, which is when the WHO declared COVID-19 as a pandemic. Associations between the 2 groups and the risk of RSV infection were also estimated using risk differences and confidence intervals. Statistical significance was set at *P* < .05, and the analyses were not adjusted for multiple comparisons. All data were analyzed using Stata 17.0 (StataCorp LP, College Station, TX).

## 3. Results

### 3.1. Study population

**Figure 1.**
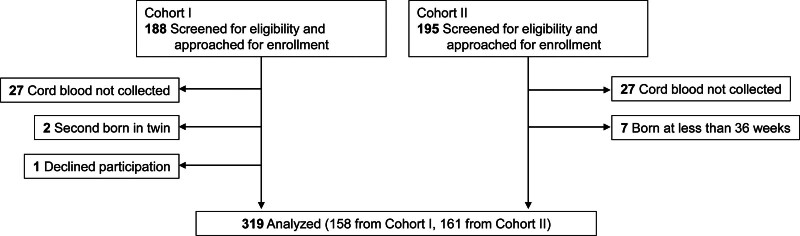
Diagram showing participant flow in the study.

A total of 383 mother–infant pairs, comprising 188 from Cohort I and 195 from Cohort II, were eligible for the study, among whom 319 pairs were enrolled (Fig. 1). The reasons for non-enrollment included missing cord blood samples (n = 54), births before 36 weeks gestation (n = 7), twin births (n = 2), and declined participation (n = 1).

Participants’ characteristics were compared between infants born in 2019 (n = 109), 2020 (n = 27), 2021 (n = 22), and 2022 (n = 161) (Table 1). Mean maternal age [SD] was 35.4 [4.8] years. A total of 117 women (36.7%) conceived through assisted reproductive technology; 107 infants (33.5%) were born via cesarean section. The median [IQR] gestational age at delivery was 39 [38–39] weeks, mean birthweight was 3004.5 [426.2] g. The characteristics of mothers and newborns pre-pandemic year 2019 versus during the pandemic year 2022 did not differ, except for the number of siblings. The number of participants with siblings was 6 (5.5%) in 2019 and 74 (46.0%) in 2022.

**Table 1:** Characteristics of mothers and newborns.

	Childbirths, no. (%)			
	2019 (n = 109)	2020 (n = 27)	2021 (n = 22)	2022 (n = 161)
Maternal age, mean (SD), y	35.3 (4.4)	35.0 (4.7)	35.4 (3.8)	35.5 (5.2)
Nationality				
Japan	107 (98.2)	25 (92.6)	22 (100)	160 (99.4)
China	1 (0.9)	2 (7.4)	0 (0.0)	0 (0.0)
Hong Kong	0 (0.0)	0 (0.0)	0 (0.0)	1 (0.6)
Thailand	1 (0.9)	0 (0.0)	0 (0.0)	0 (0.0)
Education attainment				
High school or less	2 (1.8)	1 (3.7)	0 (0.0)	8 (5.0)
College degree	24 (22.0)	1 (3.7)	4 (18.2)	22 (13.7)
University degree	68 (62.4)	21 (77.8)	17 (77.3)	97 (60.2)
Graduate degree	9 (8.3)	4 (14.8)	1 (4.5)	19 (11.8)
Unknown	6 (5.5)	0 (0.0)	0 (0.0)	15 (9.3)
Method of conception				
Natural	64 (58.7)	16 (59.3)	14 (63.6)	89 (55.3)
Infertility treatment without ART	4 (3.7)	1 (3.7)	0 (0.0)	14 (8.7)
ART	41 (37.6)	10 (37.0)	8 (36.4)	58 (36.0)
Pregravid BMI				
<18.5	18 (16.5)	10 (37.0)	1 (4.5)	33 (20.5)
18.5–24.9	81 (74.3)	15 (55.6)	20 (90.9)	117 (72.7)
25.0–29.9	8 (7.3)	2 (7.4)	1 (4.5)	11 (6.8)
≥30.0	2 (1.8)	0 (0.0)	0 (0.0)	0 (0.0)
Pregnancy type				
Singleton	107 (98.2)	27 (100)	22 (100)	161 (100)
Twin	2 (1.8)	0 (0.0)	0 (0.0)	0 (0.0)
Gestational weight gain, median (IQR), kg	10.0 (8.0–12.5)	10.1 (7.8–13.0)	9.6 (6.3–13.2)	10.5 (8.6–12.1)
Number of siblings				
0	103 (94.5)	24 (88.9)	7 (31.8)	87 (54.0)
1	6 (5.5)	2 (7.4)	14 (63.6)	63 (39.1)
2	0 (0.0)	1 (3.7)	1 (4.5)	7 (4.3)
3	0 (0.0)	0 (0.0)	0 (0.0)	4 (2.5)
Age of youngest sibling				
<2	0 (0.0)	1 (3.7)	6 (27.3)	9 (5.6)
2–5	5 (4.6)	2 (7.4)	8 (36.4)	57 (35.4)
≥6	1 (0.9)	0 (0.0)	1 (4.5)	8 (5.0)
Gestational age at delivery, median (IQR), weeks	39 (38–39)	39 (38–39)	39 (38–39)	39 (38–39)
Mode of delivery				
Vaginal	70 (64.2)	18 (66.7)	17 (77.3)	107 (66.5)
Cesarean	39 (35.8)	9 (33.3)	5 (22.7)	54 (33.5)
Infant sex				
Male	55 (50.5)	15 (55.6)	11 (50.0)	92 (57.1)
Female	54 (49.5)	12 (44.4)	11 (50.0)	69 (42.9)
Birthweight, mean (SD), g	3030.0 (385.6)	3017.1 (398.9)	2991.4 (293.1)	2986.9 (472.1)
Apgar score, median (IQR)				
1 minute	8 (8–8)	8 (8–9)	8 (8–8)	8 (8–8)
5 minutes	9 (9–9)	9 (9–10)	9 (9–10)	9 (9–9)

ART = assisted reproductive technology, BMI = body mass index (calculated as weight in kilograms divided by height in meters squared).

### 3.2. Cord blood IgG positivity

**Figure 2.**
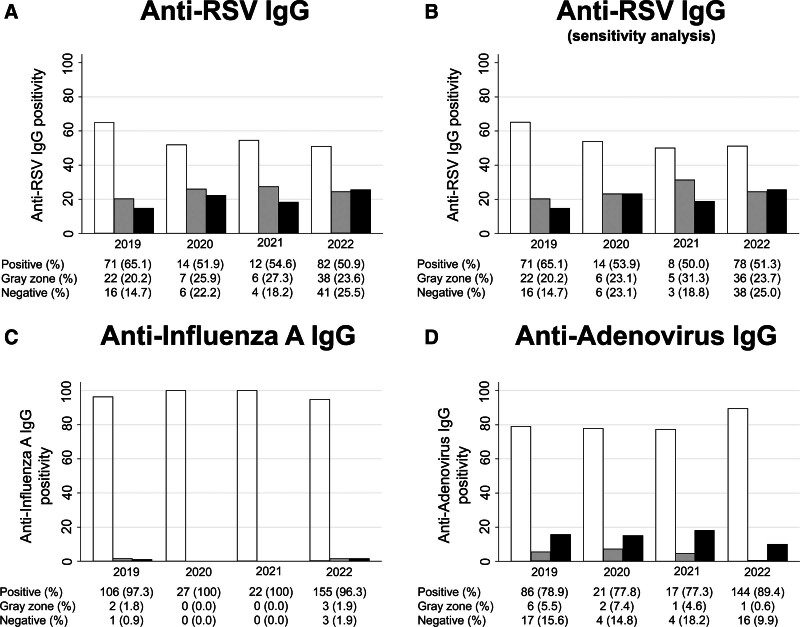
Cord blood IgG antibody levels. From 2019 to 2022, cord blood IgG positivity for RSV (A), sensitivity analysis for RSV excluding participants with siblings under 2 years (B), and cord blood IgG positivity for influenza A virus (C), and adenovirus (D) were compared. White, gray, and black boxes represent positive, gray zone and negative, respectively. IgG = immunoglobulin G, RSV = respiratory syncytial virus.

Plasma IgG positivity for RSV, influenza A and adenovirus in the 319 cord blood samples obtained at birth was compared among infants born between 2019 and 2022. In the pandemic year 2022, anti-RSV IgG tested positive in 82 of 161 cord blood samples (50.9%), which was significantly lower than in the pre-pandemic year 2019 (71 of 109 samples, 65.1%; *P* = .02) (Fig. 2A). Sensitivity analysis, performed by excluding 16 households with siblings under 2 years of age at the birth of the study infant, also showed similar and significant differences in positivity between 2019 (65.1%) and 2022 (51.3%; *P* = .03) (Fig. 2B). Conversely, anti-influenza A IgG positivity remained consistently high, with positivity ratios between 96% and 100% (Fig. 2C). Similarly, anti-adenovirus IgG positivity remained relatively high, hovering around 80% (Fig. 2D).

### 3.3. Characteristics of 5 infantile/severe RSV infection cases

We examined the association between cord blood anti-RSV IgG antibody positivity and the risk of RSV infection by the second birthday in 130 Cohort I participants with available infection information. Among the infants, 43 (33.1%) were diagnosed with RSV infections by local pediatricians, among which 5 cases (11.6% among the 43 infected infants and 3.8% among the total of 130 infants) were considered to be the ‘infantile/severe’ form (see Table S1, Supplemental Digital Content, http://links.lww.com/MD/O244 which illustrates the characteristics of moderate severity infections in infants with RSV). Among these cases, 4 were infected with RSV during infancy, before their first birthday: 2 had persistent asthmatic bronchiolitis for over a month at 2 months of age; 1 developed asthmatic bronchiolitis at 10 months, which required emergency care and over a month for recovery; the fourth infant displayed croup symptoms at 11 months, necessitating emergency care and steroid treatment. One case was infected with RSV twice, at 2 and 15 months of age. One contracted RSV at 1 year and 4 months old, with asthmatic bronchiolitis symptoms accompanied by fever, tachypnea, and hypoxia. Hospitalization, mechanical ventilation, and fatalities were not observed in this study.

### 3.4. Risk of RSV infection in relation to anti-RSV IgG status at birth: positive versus non-positive

This study examined if anti-RSV IgG positivity in cord blood at birth impacted the risk of subsequent RSV infection (Table 2). Among 80 infants with anti-RSV IgG positivity at birth, 28 (35.0%) were diagnosed with RSV infection (both mild and infantile/severe) by their second birthday, comparable to 15 (30.0%) of the 50 non-positives (negative + gray zone). All 5 (10.0%) infantile/severe cases occurred among the 50 non-positives (*P* = .004). Four infantile cases under 1 year old (*P* = .01) or 3 cases with asthmatic bronchiolitis persisting for over a month (*P* = .03) occurred in the 50 non-positive infants, with no occurrences in the 80 positives.

**Table 2:** Risk of RSV infections according to anti-RSV IgG status at birth: positive versus non-positive.

	Positive N = 80	Non-positive N = 50	RD, % (95% CI)	*P*-value
Total RSV infections (n = 43)	28 (35.0%)	15 (30.0%)	5 (-11 to 21)	.56
Infantile/severe form (n = 5)	0 (0.0%)	5 (10.0%)	-10 (-18 to -2)	.004
Onset at 2 months (n = 2)	0 (0.0%)	2 (4.0%)	-4 (-9 to 1)	.07
Infantile, in under 1-year-olds (n = 4)	0 (0.0%)	4 (8.0%)	-8 (-16 to -0)	.01
Asthmatic bronchiolitis persisting for over a month (n = 3)	0 (0.0%)	3 (6.0%)	-6 (-13 to -1)	.03

IgG = immunoglobulin G, RD = risk differences, RSV = respiratory syncytial virus.

### 3.5. Risk of RSV infection by period: pandemic versus pre-pandemic

The risks of RSV infection, both overall and considering specific characteristics, were compared between pandemic (n = 24) and pre-pandemic periods (n = 106) with the cutoff date when WHO declared the COVID-19 pandemic (Table 3). Throughout the pandemic, 8 infants (33.3%) experienced RSV infections, which did not differ significantly from the 35 cases (33.0%) observed in the pre-pandemic period. However, infantile/severe RSV infection affected 4 infants (16.7%) during the pandemic, which was a significantly higher incidence than the single pre-pandemic period case (0.9%) (*P* < .001). Similarly, the risks of RSV infection with specific characteristics: (1) onset at 2 months (*P* = .003), (2) occurring before 1 year old (*P* = .003), and (3) disease duration exceeding 1 month (*P* = .03), were also significantly higher during the pandemic as compared to the pre-pandemic period.

**Table 3:** Risk of RSV infections by period: pandemic versus pre-pandemic.

	During the pandemic N = 24	Pre-pandemic N = 106	RD, % (95% CI)	*P*-value
Total (n = 43)	8 (33.3%)	35 (33.0%)	0 (-21 to 21)	.98
Infantile/severe form (n = 5)	4 (16.7%)	1 (0.9%)	16 (1 to 31)	<.001
Onset at 2 months (n = 2)	2 (8.3%)	0 (0.0%)	8 (-3 to 19)	.003
Infantile, in under 1-year-olds (n = 4)	3 (12.5%)	1 (0.9%)	12 (-2 to 25)	.003
Disease duration, >1 month (n = 3)	2 (8.3%)	1 (0.9%)	7 (-4 to 19)	.03

CI = confidence interval, RD = risk differences, RSV = respiratory syncytial virus.

### 3.6. IgG positivity at birth and risk of influenza A and adenovirus infection

Compared to RSV, the incidence of influenza A and adenovirus infection was lower, affecting only 6 (4.6%) and 9 (6.9%) of the 130 infants in Cohort I, respectively. All cases occurred in the pre-pandemic period. Comparing IgG levels at birth between infants with and without infections, no statistically significant differences were observed for both influenza A and adenovirus infections.

### 3.7. Surveillance trends

Weekly RSV cases (ages 0 to < 2 years) in Tokyo are shown with mid-September peaks in 2018 and 2019, no distinct peak in 2020, a strong July peak in 2021, pre-pandemic-like peak in 2022, and another July peak in 2023 (see Figure S1A, Supplemental Digital Content, http://links.lww.com/MD/O244 which illustrates weekly with RSV infection cases).

Weekly influenza counts (ages 0 to <2 years) in Tokyo are also provided, demonstrating late January peaks in 2018 and 2019, shift to a mid-January peak in 2020, no noticeable peaks in 2021 to 2022, and a minor late January peak in 2023 (see Figure S1B, Supplemental Digital Content, http://links.lww.com/MD/O244 which illustrates weekly influenza infection cases).

Weekly pharyngoconjunctival fever cases (ages 0 to <2 years) in Tokyo show June peaks in 2018 to 2019, moderate prevalence in 2020 autumn to winter, and a prolonged May to July peak since 2021 (see Figure S1C, Supplemental Digital Content, http://links.lww.com/MD/O244 which illustrates weekly pharyngoconjunctival fever cases).

## 4. Discussion

In the pandemic year 2022, cord blood anti-RSV IgG positivity (50.9%) was lower compared to the pre-pandemic year 2019 (65.1%), aligning with previous reports indicating that cord blood anti-RSV IgG was positive in 60% to 80% prior to the pandemic.^[18,19]^ Even after excluding 16 households with siblings under the age of 2 years in the sensitivity analysis, the results remained consistent, demonstrating lower anti-RSV antibody levels in cord blood during the pandemic compared to pre-pandemic levels. These findings support the hypothesis that stringent COVID-19 measures might have reduced RSV exposure in pregnant women, potentially leading to a decreased transfer of anti-RSV IgG to fetuses through the placenta, consequently resulting in diminished cord blood IgG positivity.

Moreover, reduced anti-RSV IgG positivity at birth was associated with the risk of infantile/severe RSV infections, rather than the overall number of RSV infections, including mild cases. Additionally, there was a higher incidence of infantile/severe RSV infections during the pandemic compared to the pre-pandemic period. A randomized clinical trial of RSV vaccination during pregnancy reported successful prevention of medically-attended severe lower respiratory tract illnesses in infants,^[20]^ but not of medically-significant lower respiratory tract infections.^[20,21]^ The presence of anti-RSV IgG at birth diminishes by 6 months,^[18,19]^ coinciding with the greater likelihood of severe RSV infections in infants, particularly those aged 6 months and younger.^[7,9]^ These findings support the potential role of placental IgG transfer from mothers to fetuses in preventing infantile/severe RSV infections. This raises the possibility that the resurgence of RSV in Tokyo during the summer of 2021 might have been related to at least 3 factors: reduced placental IgG transfer; decreased RSV exposure among infants; and limited infant IgG production. This scenario, coupled with a susceptible older child population, could have contributed to the resurgence.^[22,23]^ Notably, the rise in hospital admissions and need for ventilation was the highest among children over 2 years of age,^[24,25]^ although this study did not cover this age group.

Unlike RSV, influenza A IgG positivity at birth remained consistently high both before and during the pandemic. In Japan, pregnant women were advised to receive influenza vaccination both before and during the pandemic. Maintenance of influenza IgG positivity at birth despite limited influenza spread after declaration of the pandemic in Tokyo, Japan and other countries^[26]^ could be attributed to effective vaccination among pregnant women. Furthermore, influenza typically prompts strong strain-specific immunity from previous infections.^[27]^ This likely accounts for the unchanged influenza A IgG positivity rate even during the pandemic, since past infections confer durable immunity. In contrast, anti-RSV IgG might last for a shorter duration and require repeated maternal exposure to the virus for optimal infant antibody positivity. Waning RSV immunity might explain its resurgence, while maintenance of robust influenza immunity might explain the absence of significant surges in infants and small children under 2 years of age during the COVID-19 pandemic.

Adenovirus IgG positivity at birth was also consistent pre- and during the pandemic. However, unlike influenza, pharyngoconjunctival fever, which is likely caused by adenovirus, displayed seasonal oscillation even during the pandemic in Tokyo. Consequently, pregnant women were possibly exposed to adenovirus during the pandemic, thereby maintaining unaltered transplacental transfer of adenovirus IgG to their fetuses.

## 5. Limitations

This study has several limitations. First, the study’s statistical power is limited due to the small number of severe RSV infections (5 cases) within the sample size of 43, which may affect the reliability of the conclusions drawn. Second, there were fewer participants during the early phases of the pandemic (e.g., 2020 and 2021) compared to the pre-pandemic period (i.e., 2019) and the later phase of the pandemic (e.g., 2022), which could potentially introduce confounding and biases in the assessment of infection risks. Third, the lack of specificity of the assay method used for detecting anti-RSV IgG antibodies in this study, particularly its inability to target the prefusion F antigen, which is crucial for effective viral neutralization, represents a notable limitation necessitating further investigation. Fourth, the incidences of RSV, influenza and adenovirus infections up to 2 years of age might possibly have been underestimated in this study. This is because mothers with febrile children might have avoided medical facilities in Tokyo during the pandemic, potentially leading to underestimation of infections. Since infection with RSV, influenza and adenovirus can be asymptomatic, cases might have gone undiagnosed, leading to further underestimation of the reported infection rates. Moreover, pediatricians might not always employ rapid antigen tests via nasopharyngeal swabs, contributing to underestimation of mild cases. Additionally, there are diagnostic accuracy concerns in relation to the testing kits used. Fifth, infantile/severe RSV infections were retrospectively defined, potentially introducing selection bias. Considering that the study outcome occurred in just 5 infants, the significant results might have been coincidental. Sixth, data on influenza immunizations during pregnancy were unavailable for analysis, although vaccination rates were considered to be high. Seventh, the number of siblings was higher during the pandemic than in the pre-pandemic period. This is because Cohort I (born between February 4, 2019 and December 31, 2021) included mostly first-time mothers pre-pandemic, while Cohort II (born between January 1, 2022 and August 31, 2022) included more second/subsequent births from 2022 onwards. However, sensitivity analysis, excluding households with siblings under 2 years old, showed consistent results, minimizing confounding. Eighth, anti-RSV IgG levels were measured in cord blood but not during the infantile period. Recent evidence showed that serum levels of anti-RSV IgG were slightly decreased in women of childbearing age a year after the COVID-19 pandemic, with substantial decline in infants probably due to decreased viral exposure.^[28]^ Nineth, this single-center study in central Tokyo involved older maternal age, over a third with in vitro fertilization, and nearly a third with cesarean births, possibly limiting its generalizability.

## 6. Conclusion

This study found an association between decreased cord blood anti-RSV IgG positivity during the COVID-19 pandemic and an elevated risk of infantile/severe RSV infections. Conversely, IgG levels for influenza A and adenovirus remained stable from pre-pandemic year 2019 to the late pandemic year 2022.

## Acknowledgments

We would like to thank the pregnant women who agreed to provide blood samples and demographic data for this research project. The authors would like to thank Ms Haruka Wada at the Division of Molecular Epidemiology, The Jikei University School of Medicine (Tokyo, Japan) for data management and data monitoring.

## Author contributions

**Conceptualization:** Ritsuko Ogasawara, Mitsuyoshi Urashima.

**Data curation:** Ritsuko Ogasawara.

**Formal analysis:** Mitsuyoshi Urashima.

**Funding acquisition:** Mitsuyoshi Urashima.

**Investigation:** Ritsuko Ogasawara.

**Methodology:** Ritsuko Ogasawara.

**Supervision:** Mitsuyoshi Urashima.

**Visualization:** Mitsuyoshi Urashima.

**Writing – original draft:** Ritsuko Ogasawara.

**Writing – review & editing:** Mitsuyoshi Urashima.

## Supplementary Material


