# Supplementary material for: Cord blood IgG for respiratory syncytial virus and subsequent infection during the COVID-19 pandemic
Source: Medicine (Baltimore). 2025 Jan 3;104(1):e41110. doi: 10.1097/MD.0000000000041110 (PMC11709157; doi:10.1097/MD.0000000000041110)
Supplement: Supplementary file 1 [file medi-104-e41110-s001.docx]

**Supplemental Digital Content 1. Methods. Study Cohort Descriptions**

**Cohort I (ABC II)**

This trial aims to clarify whether food allergies in children by 2 years of age is preventable by restricting maternal intake of processed meat, fish, and other items, such as nuts, ice-cream, butter or buttery food, e.g., croissants, biscuits, cookies, pastries, etc., during the first month after birth. The study commenced on January 19, 2019 and is still ongoing. The trial protocol was approved by the ethics committee of the Jikei University School of Medicine (Nishi-shimbashi, Tokyo, Japan) (ethics approval code: 30-022[9043]). Written informed consent was obtained from all participants. The inclusion and exclusion criteria are as shown below.

Inclusion criteria

1. Pregnant women over 20 years old.
2. Infants at a high-risk of atopic sensitization, due to at least one of their parents or siblings having atopic diseases (asthma, atopic dermatitis, food allergy, allergic rhinitis, pollen allergy, allergic conjunctivitis) in the past or present.
3. Participants who understand randomization into the two groups, the ‘restricting processed meat’ group and non-restricting group.
4. Available to visit outpatient clinic by 2 years of age.
5. Available to respond to questionnaires about health conditions, e.g., asthma diagnosed by a doctor, by 5 years of age.
6. Living in Tokyo and available to visit mother and child center outpatient clinic of Jikei University Hospital.
7. Able to visit the outpatient clinic for allergic diseases at Jikei University Hospital for care by allergy specialists in case the infant is suspected to have food allergy or any other allergy.

Exclusion criteria

1. Participating pregnant women with food allergies to eggs, nuts, meat, or milk.
2. Less than 36 weeks gestational age.
3. Unable to breastfeed for medical reasons.
4. Infants with serious conditions needing admission to the neonatal intensive care unit.
5. Infants with conditions needing admission to the Growing Care Unit and judged by the attending pediatrician as being unsuitable for inclusion in this trial.
6. Difficulty in communicating with the mother.

**Cohort II**

This prospective cohort study aims to measure soluble programmed death-ligand 1 (PD-L1) levels in maternal and cord blood, and to analyze the relationship between PD-L1 and pregnancy complications, such as gestational hypertension and preterm birth. The study commenced on October 18, 2021 and is still ongoing. The trial protocol was approved by the ethics committee of the Jikei University School of Medicine (Nishi-shimbashi, Tokyo, Japan) (ethics approval code: 33–105 [10720]). Written informed consent was obtained from all participants. The inclusion and exclusion criteria are as shown below.

Inclusion criteria

1. Over 20 years of age.
2. Pregnant women scheduled to deliver at the Jikei University who meet either criteria (1) or (2) below:
3. All pregnant women undergoing medical checkups at Jikei University Hospital from less than 12 weeks' gestation.
4. Pregnant women who change the delivery site from another hospital to Jikei University Hospital between 12 weeks 0 days and 28 weeks 6 days gestation due to maternal or fetal complications.

Exclusion criteria

1. Younger than 20 years of age.
2. Multiple pregnancies.
3. Hemoglobin less than 9.0 g/dL at the time of consent.

**Supplemental Digital Content 2.**

**Table.** **Characteristics of infants with moderate severity RSV infection**

|  | **Birth year/month** | **Cord blood anti-RSV IgG at birth [SU]** | **Age (months) at which RSV infection occurred** | **Clinical course** |
| --- | --- | --- | --- | --- |
| 1 | 2019/8 | 10.66 | 10 | Symptoms of asthmatic bronchiolitis persisted for more than one month |
| 2 | 2020/3 | 8.26 | 11 | Presented with croup symptoms and was administered steroid medication |
| 3 | 2021/3 | 10.13 | 2 | Symptoms of asthmatic bronchiolitis persisted for more than one month |
| 4 | 2021/4 | 10.64 | 2 and 15 | Symptoms of asthmatic bronchiolitis persisted for more than one month during the 1^st^ episode |
| 5 | 2021/4 | 8.51 | 16 | Fever, tachypnea, hypoxia |

**Supplemental Digital Content 3. Figure 1.** Number of cases reported weekly in patients aged 0 to <2 years in Tokyo --- pages 2-4

**
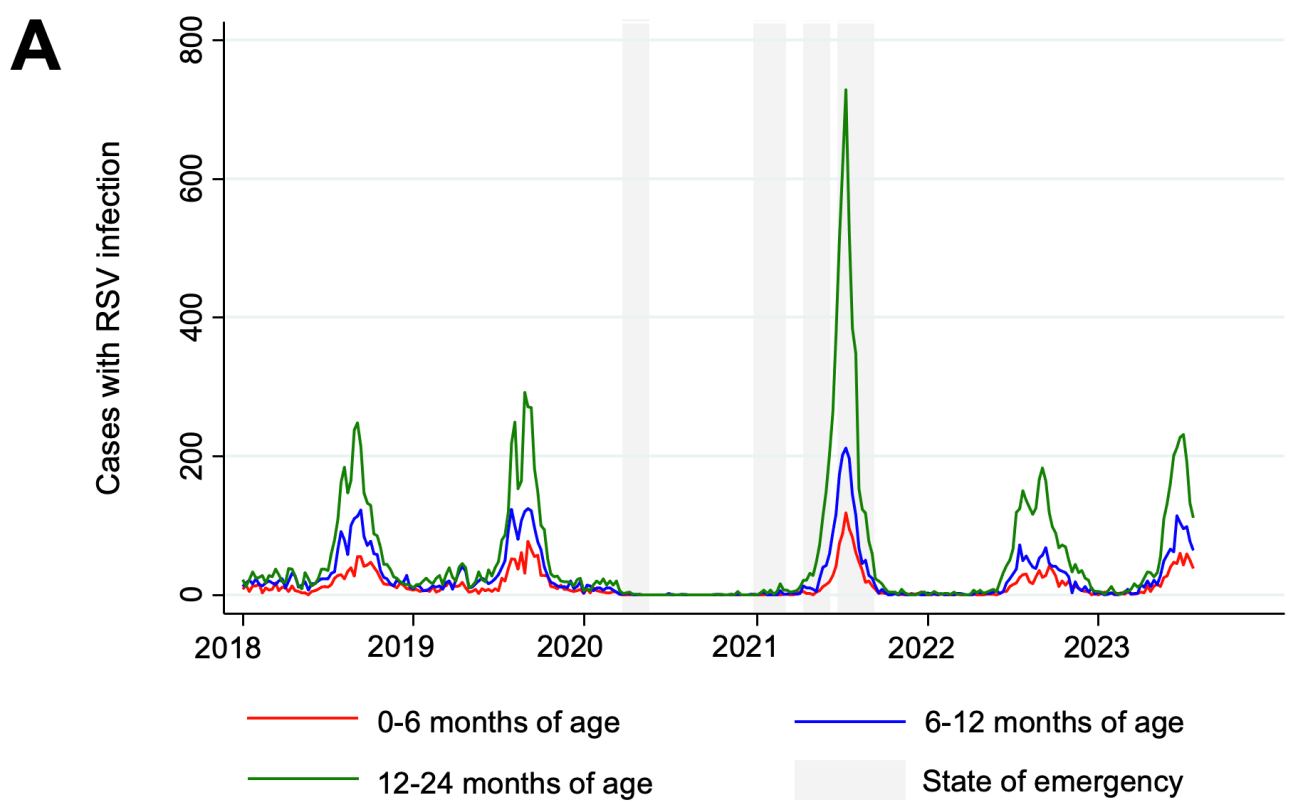
**


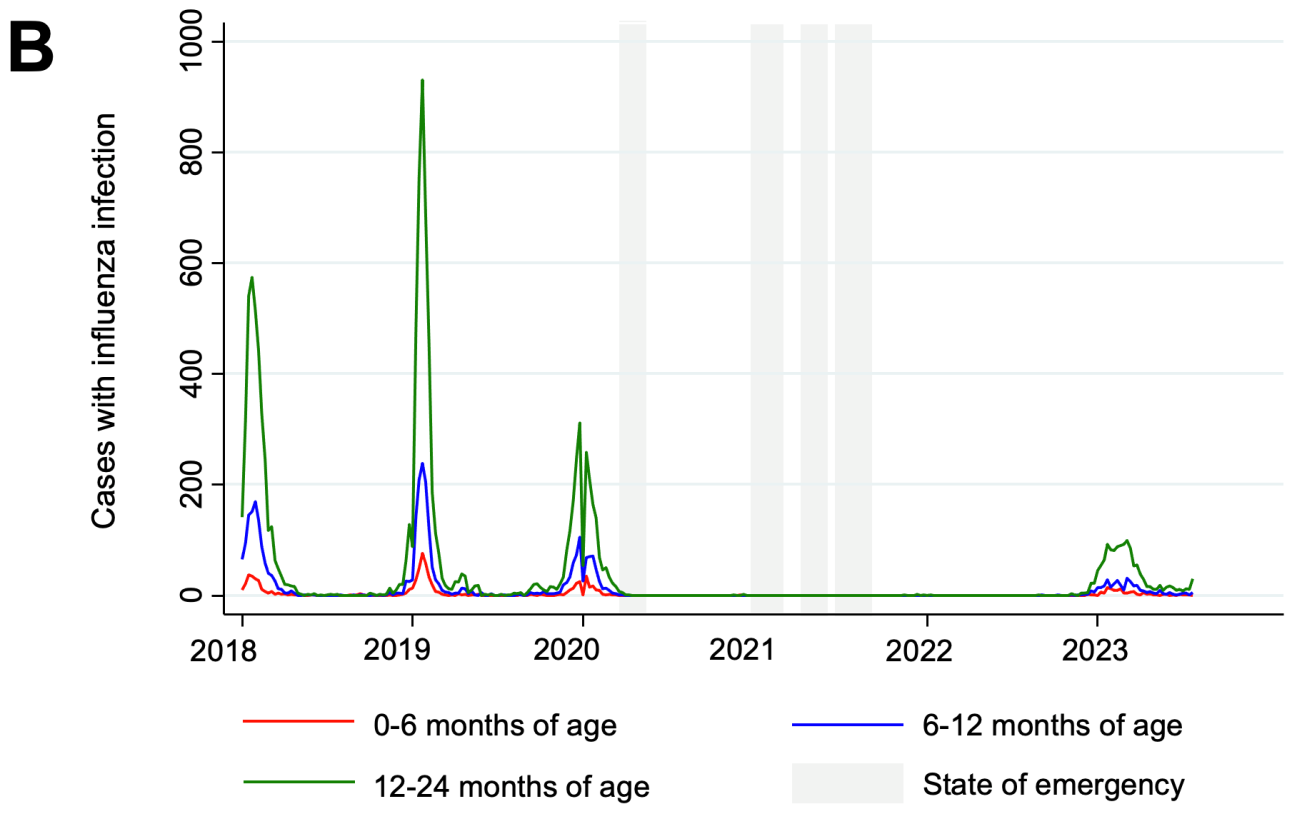


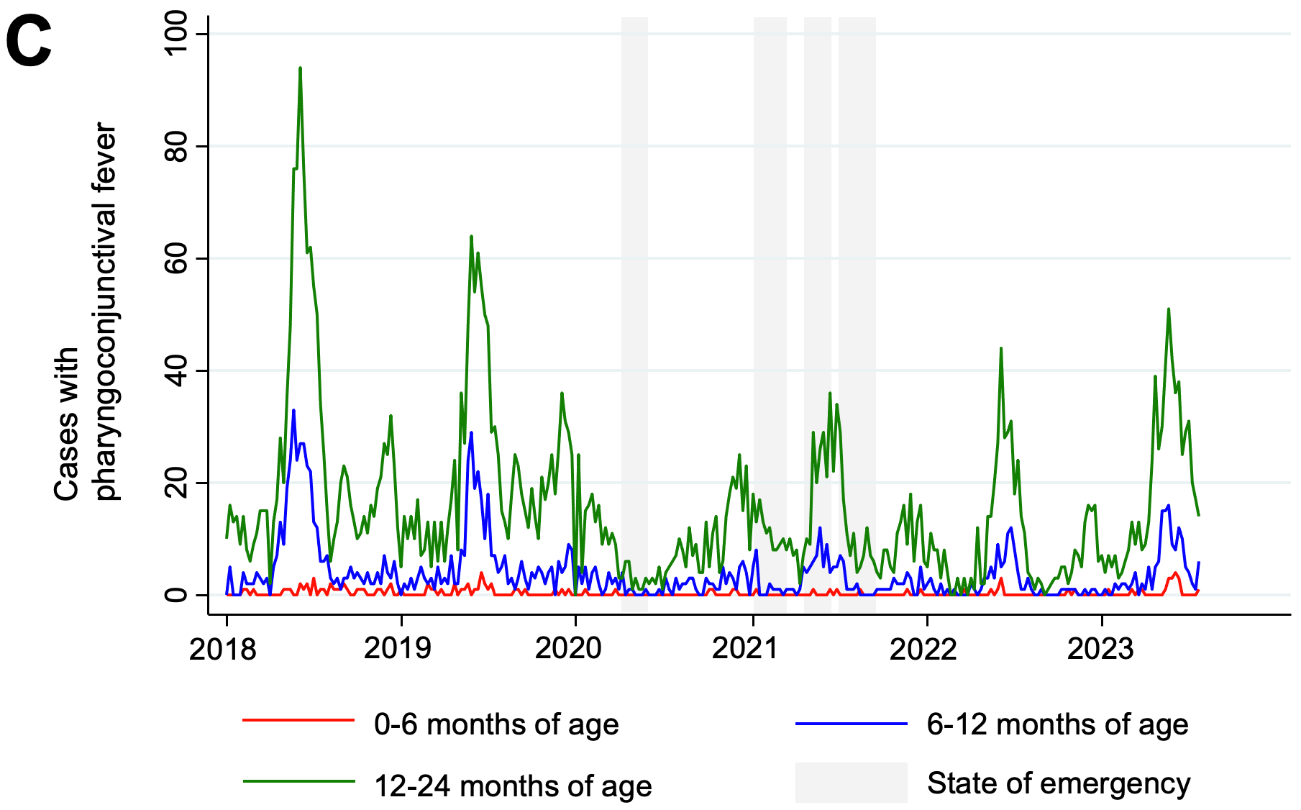


**Supplemental Digital Content 3. Figure 1. Number of cases reported weekly in patients aged 0 to <2 years in Tokyo**

RSV infections (A), influenza (B), and pharyngoconjunctival fever (C) in children under 24 months of age, by year and epidemiological week, in Tokyo, Japan, from January 1, 2019 to July 31, 2023. In Tokyo, a state of emergency was declared four times: from April 7, 2020 to May 25, 2020; from January 8, 2021 to March 21, 2021; from April 25, 2021 to June 20, 2021; and from July 12, 2021 to September 30, 2021. A state of emergency was declared when it was determined that the rapid spread of COVID-19 was having a serious impact on the lives and health of people and was significantly affecting daily life and the national economy.
